# Primary Ciliary Dyskinesia Diagnostic Challenges: Understanding the Clinical Phenotype of the Puerto Rican *RSPH4A* Founder Mutation

**DOI:** 10.3390/diagnostics11020281

**Published:** 2021-02-11

**Authors:** Wilfredo De Jesús-Rojas, Dalilah Reyes-De Jesús, Ricardo A. Mosquera

**Affiliations:** 1 Department of Pediatrics, Medical Sciences Campus, School of Medicine, University of Puerto Rico, San Juan 00921, Puerto Rico; dalilah.reyes@upr.edu; 2 Department of Pediatrics, Houston Medical School, University of Texas Health Science Center, Houston, TX 77030, USA; ricardo.a.mosquera@uth.tmc.edu

**Keywords:** *RSPH4A*, primary ciliary dyskinesia, founder mutation, cilia, Puerto Rico

## Abstract

Primary ciliary dyskinesia (PCD) is a rare, heterogeneous ciliopathy resulting in chronic oto-sino-pulmonary disease, bronchiectasis, newborn respiratory distress, and laterality defects. PCD diagnosis can be achieved by following diagnostic algorithms that include electron microscopy, genetics, and ancillary testing. Genetic mutations in more than 45 genes, including *RSPH4A*, can lead to PCD. *RSPH4A* mutations located on chromosome six, affect radial spokes and results in central complex apparatus abnormalities. The *RSPH4A* [c.921 + 3_6delAAGT] founder mutation was described as one cause of PCD without laterality defects in Puerto Rico. Additionally, there are further diagnostic challenges present in the Puerto Rican population to diagnose PCD. We describe the demographics, clinical features, and *RSPH4A* genetic variants in 13 patients with clinical PCD affecting 11 Puerto Ricans from unrelated families.

## 1. Introduction

Primary ciliary dyskinesia (PCD) is a genetically heterogeneous, autosomal recessive disorder characterized by motile cilia dysfunction with a prevalence of approximately 1:15,000 individuals in the United States [1]. The actual prevalence of PCD in Puerto Rico is unknown. Genetic mutations in more than 45 human genes can lead to PCD, affecting the function of around 200 ciliary structural proteins [2]. These abnormalities result phenotypically in neonatal respiratory distress in approximately 80% despite a full-term gestation, oto-sino-pulmonary disease since birth, infertility, and organ laterality defects in 40–50% of cases [3]. Diagnostic algorithms for suspected PCD patients recommend evaluation of individuals with at least 2 of the 4 key clinical features including unexplained neonatal respiratory distress in term infants, year-round daily cough and/or daily nasal congestion beginning before 6 months of age, and laterality defects [4,5].

PCD diagnosis has become a challenge for physicians since it is easily misdiagnosed with similar genetic syndromes such as cystic fibrosis and other chronic suppurative lung diseases [6]. Previous studies have shown that approximately 1 of 3 patients have a normal ciliary structure on electron microscopy, which increases the difficulty in confirming the PCD diagnosis [7]. As a key diagnostic test, electron microscopy evaluates the 9 + 2 ciliary normal configuration and assesses for ciliary ultrastructure defects. Updated guidelines incorporate genetic testing as part of the algorithm for PCD diagnosis [4]. Additional PCD mutations have been discovered as genetic testing has become more accessible to healthcare providers and patients. 

Biallelic pathogenic variants in the *RSPH4A* gene, located on chromosome 6, affect ciliary radial spokes which alter the configuration of the central complex apparatus [8]. Mutations in *RSPH4A* affect the normal planar beating pattern of the cilia into a circular ciliary motion [9]. The abnormal ciliary function impairs effective mucociliary clearance in PCD patients [10]. PCD mutations may interfere with the placement of the left-right organ, which is established during embryogenesis [11]. Normally, the embryonic node cilium lacks a central pair of microtubules following a 9 + 0 axonemal structure, which specifies the left-right axis [12]. Therefore, PCD mutations that affect radial spokes, like *RSPH4A*, will not present laterality defects like dextrocardia or complete situs inversus [13]. *RSPH4A* [c.921 + 3_6delAAGT] founder mutation was described as a PCD pathogenic variant without laterality defects. The splice site mutation [c.921 + 3_6delAAGT] in *RSPH4A* has been described as a common cause of PCD in the Hispanic population, specifically Puerto Rico [14]. We aim to describe our experience with clinical features, pulmonary function test, chest imaging, genetic profile, and electron microscopy findings diagnosing native Puerto Ricans with *RSPH4A* mutations. A better understanding of the clinical-genetic correlation of *RSPH4A* genetic variants will help to expose the diagnostic challenges for PCD patients without laterality defects in Puerto Rico.

## 2. Materials and Methods

A descriptive retrospective chart review of 31 Puerto Rican subjects was completed. Patients were followed up at a single private outpatient clinic in Puerto Rico with expertise in PCD from September 2018 to September 2020. Detailed histories, physical examinations, pulmonary function tests, imaging studies, and nasal ciliary biopsies were completed. Criteria for PCD evaluation were achieved by documenting the presence of 2 of the 4 key clinical features described on the suggested diagnostic algorithm as previously published [5]. Measurement of nasal nitric oxide (nNO) was not available in our institution. The sweat test was completed to rule out cystic fibrosis. Genetic testing was evaluated using commercially available sequence analysis (Invitae Corporation, San Francisco, CA, USA), using buccal swabs or saliva samples. Gene panel testing evaluated for deletion/duplication on 35 PCD-related genes. The cystic fibrosis transmembrane conductance regulator (CFTR) gene was also evaluated as part of the initial patient PCD evaluation. Genetic testing included a sequence of exons and introns for the *RSPH4A* gene (Transcript: NM_001010892.2). Nasal biopsies were submitted for pathology review at Focus Pathology Medical Laboratories in Chester, New York. A total of thirteen (n = 13) PCD patients with the presence of *RSPH4A* mutations were included in the analysis. High-Resolution chest CT (HRCT) scans were reviewed, if available. Pulmonary function tests were completed in accordance with the American Thoracic Society guidelines [15]. Airflow limitation was determined using Z-score values of −1.64 as the lower limit of normal. The Global Lung Function Initiative-2012 (GLI) was used as a reference equation. Descriptive statistics presented in percentages and means. The Institutional Review Boards approved data collection and analysis for the Protection of Human Subjects from University of Puerto Rico, Medical Sciences Campus in San Juan, Puerto Rico.

## 3. Results

### 3.1. Demographics and Clinical Characteristics

Thirteen patients had clinical PCD phenotype, at least 2 of the key clinical features for PCD evaluation, presence of *RSPH4A* genetic variants, and normal or abnormal electron microscopy. Ages ranged from 1 year to 51 years old and 62%, (8/13) were females. All patients were of Hispanic ethnicity (100%, 13/13). PCD related symptoms in our cohort included: Year-round wet cough (100%, 13/13), neonatal respiratory distress (69%, 9/13), bronchiectasis (89%, 8/9), Year-round daily nasal congestion (100%, 13/13), chronic secretory otitis media (30%, 4/13), and hearing loss (69%, 9/13). In our cohort, only two females homozygous for *RSPH4A* [c.921 + 3_6delAAGT] were sexually active and self-reported to be diagnosed with infertility. Laterality defects were present in 0%, (0/13) of the subjects with *RSPH4A* mutations (Table 1 and Table A1).

### 3.2. Electron Microscopy Findings

**Figure 1:**
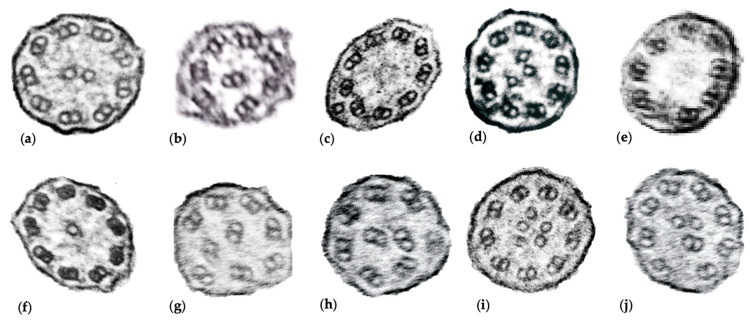
Ciliary biopsy findings of *RSPH4A* subjects. A total of 11 samples were submitted for electron microscopy, representative images of abnormal findings are shown. (**a**) Normal ciliary configuration [9 + 2]; (**b**) Abnormal peripheral microtubule configuration, [7 + 2]; (**c**) Single microtubule outside the 9 outer doublet rings [9 + 1]; (**d**) Extra central microtubule, [9 + 3]; (**e**) Electron dense material in central area surrounded by eight outer doublets [8 + 0]; (**f**) Single microtubule in position of the central pair [9 + 1]. (**g**,**h**) Abnormal microtubule organization and configuration, with displacement of peripheral doublets [9 + 0]; (**i**) Supernumerary central microtubules, [9 + 5]. (**j**) Central displacement of a peripheral doublet [8 + 4].

Ciliary biopsies were abnormal in 100%, (11/11). Abnormal findings on electron microscopy were related to central apparatus defects and abnormal microtubule configuration, including loss of central and outer doublets, presence of central solitary microtubule, and extra gain of central singlets, triplets, and quadruplets (Figure 1).

### 3.3. RSPH4A Genetic Sequencing

*RSPH4A* genetic variants were identified in all 13 subjects from 11 unrelated families. Biallelic pathogenic variants in *RSPH4A* gene were noted in 62%, (8/13) subjects of the cohort (Table A1). Rare *RSPH4A* genetic variants classified as a variant of unknown significance (VUS) were present in 23%, (3/13) of the cohort. Most common *RSPH4A* pathogenic variants were the *RSPH4A* [c.921 + 3_6delAAGT]. A single heterozygous copy of the pathogenic genetic variant *RSPH4A* [c.1103T > G] (p. Val368Gly) was present in one patient. Two subjects from unrelated families had one heterozygous copy of the *RSPH4A* [c.902A > C (p.Gln301Pro)], classified as a VUS. These two rare genetic variants have been previously described on population databases: *RSPH4A* [c.1103T > G] (p.Val368Gly); (rs747419302, ExAC 0.001%) and *RSPH4A* [c.902A > C (p.Gln301Pro)] (rs755128358, ExAC 0.009%). Both genetic variants have been reported in the literature on individuals with *RSPH4A*-related disease [www.lovd.nl/RSPH4A] (accessed on 19 February 2021). 

### 3.4. Imaging

**Figure 2:**
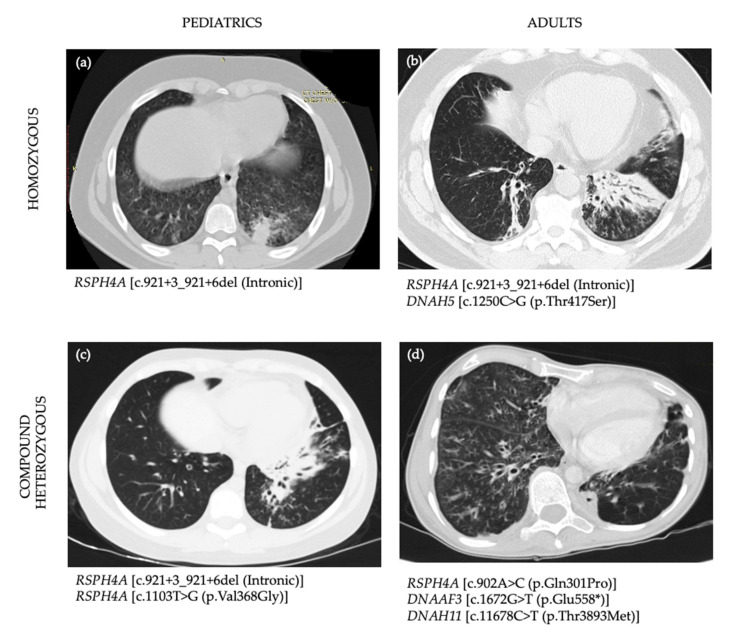
High-Resolution chest CT scans (HRCT) of PCD patients with positive *RSPH4A* genetic variants. (**a**) 15-year-old male homozygous for *RSPH4A* [c.921 + 3_921 + 6del (Intronic)] founder mutation shows a tree-in-bud pattern at left lower lobe (LLL), lingula, right lower lobe (RLL), right middle lobe (RML), right upper lobe (RUL). HRCT showed bilateral ground-glass appearance but no bronchiectasis. (**b**) 51-year-old male, homozygous for *RSPH4A* [c.921 + 3_921 + 6del (Intronic)] founder mutation who also had a heterozygous Variant of Unknown Significance (VUS) in *DNAH5* c.1250C > G (p.Thr417Ser). HRCT showed bibasilar bronchiectasis and parenchymal scarring tissue more evident at the LLL. (**c**) 13-year-old male with two pathogenic heterozygous genetic variants in *RSPH4A* [c.921 + 3_921 + 6del (Intronic)] and *RSPH4A* [c.1103T > G (p.Val368Gly)] and one heterozygous VUS in *DNAH8* [c.9839A > T (p.Gln3280Leu)]. HRCT showed cylindrical and varicose bilateral bronchiectasis, multiple centrilobular nodules, and tree-in-bud opacities. (**d**) A 33-year-old female with one heterozygous *RSPH4A* [c.902A > C (p.Gln301Pro)] VUS who showed extensive bilateral bronchiectasis, reticular-nodular infiltrates with tree-in-bud changes at RML and RLL. Case 4 also had an heterozygous VUS on *DNAAF3* [c.1672G > T (p.Glu558*)] and *DNAH11* [c.11678C > T (p.Thr3893Met)]. All four cases had central apparatus abnormalities on the cilia ultrastructure examined by electron microscopy.

HRCT were available for review in a total of 9 of 10 patients. Homozygous *RSPH4A* [c.921 + 3_6delAAGT] patients displayed bronchiectasis on 100% (8/8) of those above age seven. Case 9 and 10 with compound heterozygous genetic variants for *RSPH4A* displayed bronchiectasis on HRCT. The most common findings on HRCT were: cylindrical and varicose bilateral bronchiectasis, bibasilar parenchymal scarring tissue, centrilobular nodules, and tree-in-bud opacities. Representative views of the HRCT of both pediatric and adult PCD patients who are homozygous, compound heterozygous, or VUS for *RSPH4A* are presented in Figure 2.

### 3.5. Pulmonary Function Profile

Pulmonary function test was reviewed on subjects above age 5. Airflow limitation was noted abnormal in a total of 78%, (7/9). Obstructive and restrictive ventilatory defects were present in 55%, (5/9) and 22%, (2/9) of patients, respectively as defined by spirometry classification. Post bronchodilator challenge was positive (increase in [Forced Expiratory Volume in 1 s], FEV1 > 12%) in 40%, (2/5) of those patients with baseline airway flow obstruction. Residual volume (RV), RV/TLC, and specific airway resistance (sRaw) were noted elevated in 100%, (5/5) of patients.

## 4. Discussion

Diagnostic challenges in rare lung disorders like PCD could be numerous depending on the prevalence, clinical phenotype, and genetic variability of the population to be studied. In Puerto Rico, there is a lack of information about rare lung diseases, including PCD. The actual prevalence of PCD on the island is unknown. Considering a prevalence of PCD of 1:15,000 and the actual population of Puerto Rico to be around 2.8 million individuals, we can estimate a population of 187 patients living with PCD on the island. This estimation doesn’t count the presence of a founder genetic mutation on the *RSPH4A* gene previously described [14], which may contribute to an increased amount of patients living in Puerto Rico with PCD. The fact that in 24 months our clinic was able to diagnose a total of 13 patients with *RSPH4A*, shows that PCP in Puerto Rico has been underdiagnosed. We understand that genetic testing and electron microscopy are limitations for the Puerto Rican population to follow the established diagnostic algorithms as recommended by the PCD foundation in the United States. The lack of nNO test as a screening and diagnostic PCD tool for Puerto Rico and the Caribbean are limitations in some challenging cases. The genetic variability of the Puerto Rican population introduces additional challenges as commercial genetic testing panels with 35 genes may not be able to unmask rare PCD genetic variants as pathogenic to confirm the diagnosis when more than 45 PCD genes have been described. For instance, VUS genetic variants may need to be analyzed taking into consideration the clinical phenotype of PCD patients in Puerto Rico. To date a total of 36 cases are reported on LOVD3 (Leiden Open Variation Database), (www.lovd.nl/RSPH4A (accessed on 19 February 2021)) for *RSPH4A* mutations. A total of 17 unique cases are reported for the *RSPH4A* [c.921 + 3_6delAAGT] mutation with Puerto Rican heritage. We add a total of new 13 *RSPH4A* cases and the other 11 *RSPH4A* [c.921 + 3_6delAAGT] unique mutations from native Puerto Ricans.

In this article, we describe 13 patients, from 11 unrelated families, who were evaluated for PCD and presented *RSPH4A* mutations on genetic testing. Although 8 of 13 (62%) patients were positive for the presence of homozygous genetic mutations on *RSPH4A* [c.921 + 3_6delAAGT], 5 of 13 patients showed uncertain results, classified as VUS. While we know that *RSPH4A* [c.921 + 3_6delAAGT] genetic mutation has been detected on Hispanics living in the United States with Puerto Rican ancestry, our study confirms this observation in native Puerto Ricans with clinical PCD symptoms. Interestingly, patients with biallelic pathogenic variants in *RSPH4A* [c.921 + 3_6delAAGT] mutation showed most of the clinical PCD features including neonatal respiratory distress at birth, bronchiectasis, and sinus diseases as the main phenotype. However, chronic secretory otitis media was presented only in 33%, (2/6) of individuals with biallelic homozygous *RSPH4A* [c.921 + 3_6delAAGT] mutation. As compared with other genetic variants published [14,16], we saw fewer chronic secretory otitis media cases. Central complex defects on electron microscopy have been associated with severe otologic features in children with PCD [17]. Our results showed that central complex defects were present in 100% of the cohort. However, the severity of otologic features was not as prevalent as expected. Laterality defects were absent in our cohort as previously documented on mutations that affect the central complex apparatus. We know that *RSPH4A* mutations are not necessarily a requirement for abnormal left-right axis orientation [12].

The genetic diversity of Puerto Rican ancestry may explain the range and combination of VUS genetic variants found. Three patients (Cases 4, 7 and 8) were “purely” homozygous for the *RSPH4A* [c.921 + 3_6delAAGT] mutation. All other patients had a combination of pathogenic *RSPH4A* with other PCD related genetic heterozygous VUS. The interactions of VUS with pathogenic PCD variants are unknown. Additional studies are needed to explore the role of digenic inheritance in PCD as seen in other ciliopathies [18]. On the other hand, we show in Table A1, four cases classified as compound heterozygous genetic variants with clinical criteria for PCD. Initially, on Case 9, PCD genetic tests revealed one pathogenic variant at the *RSPH4A* [c.921 + 3_6delAAGT] mutation. Additional one likely pathogenic variant was noted: *RSPH4A* [c.1103T > G (p.Val368Gly)] plus a VUS on *DNAH8* [c.9839A > T (p.Gln3280Leu)]. Familial PCD genetic studies on Case 7 showed that *RSPH4A* [c.1103T > G (p.Val368Gly)] variant was maternally inherited. No paternal *RSPH4A* or *DNAH8* variants were detected. As a result of the family analysis, genetic reclassification was made for a VUS in *RSPH4A* [c.1103T > G (p.Val368Gly)] PCD genetic variant as pathogenic. It is important to fully understand the interaction of multiple heterozygous variants in the clinical phenotype of patients with PCD. Analysis of familial studies plays an important role in the reclassification of VUS PCD genetic variants to get a point score threshold for pathogenicity. 

Another atypical patient is presented in Case 10, which exhibited two of the main features: daily wet cough and year-round daily nasal congestion but lack laterality defects or recurrent secretory otitis media. Electron microscopy findings showed central apparatus defects and HRCT revealed extensive bilateral bronchiectasis, reticular-nodular infiltrates with tree-in-bud changes at RML and RLL (Figure 2c). Pulmonary function test demonstrated severe airflow obstruction with an FEV1: 24%. Genetic testing resulted in three VUS genetic variants including *RSPH4A*, *DNAAF3*, and *DNAH11*. Although none of these genetic VUS had been associated with PCD pathogenic phenotype; the medical history, clinical findings, electron microscopy, and the severity of her pulmonary disease evidenced by pulmonary function test and imaging, highly suggest PCD. Ancillary testing like nNO, if available, will help to confirm challenges in PCD cases like this. Two rare *RSPH4A* variants were detected in our cohort. First, the *RSPH4A* [c.1103T>G], which replace a valine with glycine at the codon 368 of the *RSPH4A* protein. This missense mutation found in Case 9 promotes physicochemical differences between both amino acids. Although the effect of this missense mutation is unclear, this variant is currently classified as pathogenic and seen in individuals with PCD phenotype. The second variant found on Case 10 is *RSPH4A* [c.902A > C], which replace glutamine with proline at the codon 301. This missense mutation was not previously reported on individuals with *RSPH4A*-related diseases and is currently classified as VUS. Additional testing and evaluation including nNO and electron microscopy will be needed to confirm the diagnosis of PCD.

## 5. Conclusions

In Puerto Rico, PCD diagnosis remains under-recognized by the general population and healthcare providers. To understand the presence of a PCD founder mutation on the island, it is important to address the medical history of recurrent pulmonary infections, bronchiectasis, and sinus disease during the initial encounter. Although asthma may be a concomitant disease in PCD, a detailed history of PCD symptoms is vital in those with severe persistent asthma not responding to standard care. Referral to knowledgeable medical doctors with PCD expertise is imperative for early patient diagnosis that will prevent or delay additional PCD comorbidities later in life. Awareness about PCD clinical features on the island must be a priority for early recognition of potential PCD cases. Collaboration among neonatologists, otorhinolaryngologists, pediatricians, infertility clinics, pulmonologists, and radiologists, among other providers, is essential to reveal the actual prevalence of PCD on the island. The presence of a founder mutation in *RSPH4A* warrants the development of a multidisciplinary center for PCD for the evaluation of suspected cases in Puerto Rico. The development of a PCD center is urgently needed to explore new founder mutations in the genetic pool of individuals living in the Caribbean and Latin-America using state-of-the-art diagnostic tools following ATS and PCD foundation diagnosis guidelines. Collaboration with PCD Centers in the United States is important considering that, to date, we do not have the capacity for lung transplantation in Puerto Rico and lung transplantation in Latin-America is limited.

## Figures and Tables

**Table 1:** Demographics and clinical characteristics.

Characteristics	Value (N = 13)
Gender (F/M), N (%)	8 (62)
Age, median ± SD, (years)	13 ± 15.2
Ethnicity, N (%), Hispanics, Puerto Ricans	13 (100)
PCD-related symptoms/sign N (%)	-
Year-round wet cough	13 (100)
Year-round daily nasal congestion	13 (100)
Neonatal respiratory distress	9 (69)
Hearing loss	9 (69)
Bronchiectasis	8/9 (89)
Chronic secretory otitis media	4 (30)
Infertility	2 (15)
Laterality defects	0 (0)

F: Female; M: Male, SD: Standard Deviation.

## Data Availability

All data analyzed during this study are included in this published article.

## Abbreviations

PCD	Primary ciliary dyskinesia
nNO	nasal nitric oxide
HRCT	High-resolution chest computer tomography
CFTR	Cystic fibrosis transmembrane conductance regulator
GLI	Global lung function initiative-2012
LLL	Left lower lobe
RLL	Right lower lobe
RML	Right middle lobe
RUL	Right upper lobe
VUS	Variant of unknown significance
FEV1	Forced Expiratory Volume in 1 second
RV	Residual Volume
TLC	Total Lung Capacity
sRAW	Specific airway resistance
RDS	Respiratory Distress Syndrome

## Appendix A

**Table A1:** Demographics and PCD Genetic Variants in 13 PCD patients from Puerto.

Case	Sex	Age (y)	Laterality Defects	Ciliary Defects	Ethnicity	Chronic Cough	Neonatal RDS	Bronchiectasis on HRCT	Chronic Rhinorrhea	Chronic SecretoryOM	Hearing Loss	FEV1 (%)	Affected PCD Gene	Base Change	Amino-Acid Change	Zygocity	Classification
**Homozygous Genetic Variants**																	
**1**	M	1	0	CA	Hispanic	Y	Y	-	Y	N	Y	-	*RSPH4A* *CCDC151* *DNAH5*	c.921 + 3_921 + 6del c.619C > T c.1250C > G	Intronic p.Arg207Trp p.Thr417Ser	Homo Hete Hete	Pathogenic VUS VUS
**2**	F	7	0	R*	Hispanic	Y	Y	Y	Y	N	Y	106	*RSPH4A* *DNAH11*	c.921 + 3_921 + 6del c.3133C > T	Intronic p.Arg1045*	Homo Hete	Pathogenic Pathogenic
**3**	F	11	0	CA	Hispanic	Y	Y	Y	Y	Y	Y	79	*RSPH4A* *CCDC114* *DNAH11* *GAS8*	c.921 + 3_921 + 6del c.203C > T c.8093T > C Deletion	Intronic p.Ala68Val p.Leu2698Ser Exons 9-11	Homo Hete Hete Hete	Pathogenic VUS VUS VUS
**4**	M	15	0	CA	Hispanic	Y	Y	Y	Y	Y	Y	72	*RSPH4A*	c.921 + 3_921 + 6del	Intronic	Homo	Pathogenic
**5**	M	51	0	CA	Hispanic	Y	Y	Y	Y	N	Y	62	*RSPH4A* *DNAH5*	c.921 + 3_921 + 6del c.1250C > G	Intronic p.Thr417Ser	Homo Hete	Pathogenic VUS
**6**	F^#^	35	0	CA	Hispanic	Y	Y	Y	Y	N	Y	36	*RSPH4A* *DNAAF2*	c.921 + 3_921 + 6del c.58G > C	Intronic p.Val20Leu	Homo Hete	Pathogenic VUS
**7**	F	26	0	CA	Hispanic	Y	Y	-	Y	N	-	53	*RSPH4A*	c.921 + 3_921 + 6del	Intronic	Homo	Pathogenic
**8**	F	19	0	CA	Hispanic	Y	N	Y	Y	N	Y	59	*RSPH4A*	c.921 + 3_921 + 6del	Intronic	Homo	Pathogenic
**Compound Heterozygous Genetic Variants**																	
**9**	M	13	0	CA	Hispanic	Y	Y	Y	Y	Y	Y	60	*RSPH4A* *RSPH4A* *DNAH8*	c.921 + 3_921 + 6del c.1103T > G c.9839A > T	Intronic p.Val368Gly p.Gln3280Leu	Hete Hete Hete	Pathogenic Pathogenic^ VUS
**10**	F^#^	33	0	CA	Hispanic	Y	N	Y	Y	N	-	24	*RSPH4A* *DNAAF3* *DNAH11*	c.902A > C c.1672G > T c.11678C > T	p.Gln301Pro p.Glu558* p.Thr3893Met	Hete Hete Hete	VUS VUS VUS
**11**	F	5	0	-	Hispanic	Y	N	N	Y	Y	Y	-	*RSPH4A* *CCDC65* *DNAH1* *DNAH11*	c.921 + 3_921 + 6del c.771G > T c.9646C > G c.3288G > A	Intronic p.Glu257Asp p.Leu3216Val p.Met1096Ile	Hete Hete Hete Hete	Pathogenic VUS VUS VUS
**12**	M	2	0	CA	Hispanic	Y	Y	-	Y	N	-	-	*RSPH4A* *DNAAF4* *DNAH11* *DNAH5*	c.921 + 3_921 + 6del c.186C > G c.8888C > A c.12709G > T	Intronic p.Ser62Arg p.Ser2963Tyr p.Val4237Phe	Hete Hete Hete Hete	Pathogenic VUS VUS VUS
**Heterozygous Genetic Variants**																	
**13**	F	3	0	-	Hispanic	Y	N	-	Y	N	-	-	*RSPH4A*	c.902A > C	p.Gln301Pro	Hete	VUS

F/M: Female/Male, R*: Rare Cilia present, not diagnostic, CA: Central Apparatus defects, OM: Otitis Media, F^#^: Infertile Female, HRCT: High-Resolution Chest CT, FEV1: Forced Expiratory Volume in 1 s. RDS: Respiratory Distress Syndrome. Pathogenic^: Reclassified as pathogenic after parental genetic testing.
